# Epithelial–Mesenchymal Transition Gene Signature Related to Prognostic in Colon Adenocarcinoma

**DOI:** 10.3390/jpm11060476

**Published:** 2021-05-26

**Authors:** Constantin Busuioc, Cristina Alexandra Ciocan-Cartita, Cornelia Braicu, Oana Zanoaga, Lajos Raduly, Monica Trif, Mihai-Stefan Muresan, Calin Ionescu, Cristina Stefan, Carmen Crivii, Nadim Al Hajjar, Simona Mǎrgǎrit, Ioana Berindan-Neagoe

**Affiliations:** 1 Research Center for Functional Genomics, Biomedicine and Translational Medicine, Iuliu Hatieganu University of Medicine and Pharmacy, 23 Marinescu Street, 40015 Cluj-Napoca, Romania; busuioc.constantin@gmail.com (C.B.); crisciocan@yahoo.com (C.A.C.-C.); braicucornelia@yahoo.com (C.B.); oana.zanoaga@umfcluj.ro (O.Z.); raduly.lajos78@gmail.com (L.R.); ioananeagoe29@gmail.com (I.B.-N.); 2 Centre for Innovative Process Engineering (CENTIV) GmbH, 28857 Syke, Germany; mt@centiv.de; 3 7th Surgical Department, Iuliu Hatieganu University of Medicine and Pharmacy, 8 Victor Babes Street, 400012 Cluj-Napoca, Romania; mihai.stefan.muresan@gmail.com (M.-S.M.); ionescu_calincj2002@yahoo.com (C.I.); 4 Surgical Department, Municipal Hospital, 400139 Cluj-Napoca, Romania; 5 Sing Duke-NUS Global Health Institute Duke-NUS Medical School, 8 College Road, Singapore 169857, Singapore; cristina.stefan10@gmail.com; 6 Department of Anatomy and Embryology, Iuliu Hatieganu University of Medicine and Pharmacy, 8 Victor Babes Street, 400012 Cluj-Napoca, Romania; 7 Department of Surgery, Octavian Fodor Regional Institute of Gastroenterology and Hepatology, 19–21 Croitorilor Street, 400162 Cluj-Napoca, Romania; nadim.alhajjar@umfcluj.ro; 8 Department of Surgery, University of Medicine and Pharmacy, 19–21 Croitorilor Street, 400162 Cluj-Napoca, Romania; 9 Department of Anesthesia and Intensive Care I, Iuliu Hatieganu University of Medicine and Pharmacy, 19–21 Croitorilor Street, 400162 Cluj-Napoca, Romania; 10 Department of Intensive Care Unit, Octavian Fodor Regional Institute of Gastroenterology and Hepatology, 19–21 Croitorilor Street, 400162 Cluj-Napoca, Romania

**Keywords:** colorectal adenocarcinoma, epithelial–mesenchymal transition, prognostic markers

## Abstract

Colon adenocarcinoma (COAD) remains an important cause of cancer-related mortality worldwide. Epithelial–mesenchymal transition (EMT) is a key mechanism, promoting not only the invasive or metastatic phenotype but also resistance to therapy. Using bioinformatics approaches, we studied the alteration on EMT related genes and its implication on COAD prognostic based on public datasets. For the EMT mechanisms, two overexpressed genes were identified (NOX4 and IGF2BP3), as well as five downregulated genes (BMP5, DACT3, EEF1A2, GCNT2 and SFRP1) that were related to prognosis in COAD. A qRT-PCR validation step was conducted in a COAD patient cohort comprising of 29 tumor tissues and 29 normal adjacent tissues, endorsing the expression level for BMP5, as well as for two of the miRNAs targeting key EMT related genes, revealing upregulation of miR-27a-5p and miR-146a-5p. The EMT signature can be used to develop a panel of biomarkers for recurrence prediction in COAD patients, which may contribute to the improvement of risk stratification for the patients.

## 1. Introduction

Colon adenocarcinoma (COAD) is one of the most frequent forms of adult’s cancer type, ranking it as the third among major cancer-related death globally [1], and the most frequent form of colorectal cancer (approximately 95%) [2,3,4]. Tumor, lymph node and metastasis (TNM) staging are the standard for COAD prognostic. The prognosis for these patients is related to the TNM stage and curative surgical intervention, which is pertinent only for patients with primary tumor and loco-regional lymph nodes. Unfortunately, most of the cases are discovered in the advanced stages of the disease, when the therapeutic options are limited, being associated with a high metastatic rate and recurrence [5,6,7,8].

Epithelial to mesenchymal transition (EMT) is a mechanism, characterized by the loss of epithelial features, such as cell polarity or cell–cell contact, and acquisition of mesenchymal properties, promoting increased motility [9,10,11]. The cell–cell contact is established by tight junctions, adherent junctions, desmosomes and gap junction, being interconnected with key signaling networks [12]. Additionally, during the EMT, the epithelial actin architecture is reorganized, observing an acquisition of cell motility and invasive features and an expression of matrix metalloproteinases (MMPs) that can destroy extracellular matrix (ECM) proteins [12,13,14] and can be affected by hypoxic condition [15]. EMT is also orchestrated by several intrinsic factors, including transcription factors or miRNAs [16,17].

In COAD, similar to other cancers, the EMT mechanism is related to an invasive or metastatic phenotype [18]. EMT is a mechanism regulated by the tumor’s microenvironment components, in particular as an effect of hypoxic condition [18,19]. Therefore, investigation of the EMT mechanisms related to the COAD progression promotes the discovery of new coding and non-coding genes as diagnostic biomarkers and the development of potential powerful therapeutic target [20,21,22]. EMT is also related to chemoresistance in COAD [23,24].

Understanding the EMT key factors is vital for the progression of powerful therapeutic interventions [10]. The present study evaluates the prognostic value of the altered transcriptomic EMT signature, mRNA and microRNA (miRNA), using publicly available data of patients with COAD, followed by correlations with the EMT markers and with the related miRNAs that target these genes.

## 2. Materials and Methods

Differential gene expression analysis in COAD. We used expression data from The Cancer Omics Atlas (TCOA) repository database, which is an integrative resource for cancer omics data, allowing the user to run different types of analyses [25]. TCOA provides the inquiring of gene expression, somatic mutations, miRNA expression and protein expression data based on a single molecule or cancer type [25]. In the “Cancer” module, you are allowed to select a certain cancer type, and TCOA will further output the top 20 most frequently mutated genes, the upregulated and downregulated ones, all of them in association with the selected pathology and compared with the normal controls. We used Gene Expression Profiling Interactive Analysis (GEPIA, http://gepia.cancer-pku.cn/index.htm, accessed on 22 April 2021) for the representation of the expression level on different stages.

Pathway analyses. We generated EMT gene network analysis using String version 11.0 (https://string-db.org, accessed on 20 April 2021) [26], and an mRNA-miRNA network was generated using miRNE online tool [27,28,29].

Survival analysis. For the correlation of the survival rate for the EMT genes in COAD, GEPIA online tool was used (http://gepia.cancer-pku.cn/, accessed on 22 April 2021). Our data show only those genes able to predict the overall survival outcomes (*p*-value ≤ 0.05) in COAD. Additional survival analysis for the most relevant miRNAs targeting key EMT genes in COAD was done using StarBase [30].

Mutational pattern evaluation. The cBioPortal (http://cbioportal.org, accessed on 18 April 2021) [31] is an open-access platform that can be used for analysis of cancer genomics datasets. The EMT gene mutation pattern in COAD was obtained according to the cBioPortal’s online instructions. A mutation analysis was performed in 169 cancer studies, including mutation, amplification and deletion, based on three datasets.

Correlation among the EMT gene signature. CANCERTOOL is a friendly web-based interface that allows us to carry out gene-to-gene correlations in multiple datasets at the same time for a specific cancer subtype, including for COAD [32]. Additionally, it permits us to perform correlations among the altered genes and gene enrichment analysis. The correlation heatmap was performed based on five Affimetrix datasets (GSE44076, GSE14333, GSE33113, GSE37892, GSE39582), one Agilent dataset (GSE42284) and one RNAseq dataset. An additional correlation among the expression level of BMP5 and its related directly and indirectly interconnected miRNAs was done using the miRNA-Target CoExpression tool from StarBase (http://starbase.sysu.edu.cn/index.php, accessed on 22 April 2021) [30].

Gene and miRNAs validation in COAD samples. A total of 29 histologically confirmed COAD patients admitted were included in the study, after they signed the informant consent according to the Ethical Committee (approval number 6346/02.07.2014). Thus, the study included 17 males with age average of 70.05 ± 11.69, respectively, and 12 females with age average of 67.83 ± 11.18; these patients did not receive chemotherapy. Immediately following surgical excision, all tissue samples were snap-frozen in liquid nitrogen for RNA isolation and stored at −80 °C until further analysis. Patients’ clinical data are presented in Table 1.

Total RNA from normal and tumoral tissue was extracted and isolated according to the Trireagent (Ambion, Austin, TX, USA) protocol. The RNA concentration was measured by NanoDrop-1000 spectrophotometer (Thermo Scientific, Waltham, MA, USA). For gene expression evaluation, we used 500 ng RNA, meanwhile for miRNA, we used 50 ng. Gene expression protocol is based on a reverse transcription into cDNA step using a High-Capacity cDNA Reverse Transcription Kit (Applied Biosystems, Foster City, CA, USA), followed by a amplification step using SYBR Select Master Mix on Viia7 System, with the specific primers for target genes (BMP5: left primer: TTGTTGCCCAGGCTGGAGTG, right primer CCCAGCACTTTGGAAGGCCA; B2M: left primer CACCCCCACTGAAAAAGATGAG, right primer: CCTCCATGATGCTGCTTACATG).

The evaluation of the miRNA expression level was done using a TaqMan Based protocol and TaqMan MicroRNA Reverse Transcription Kit (Applied Biosystems). For the amplification, we used TaqMan Fast Advanced Master Mix (Applied Biosystems) and TaqMan assays (U48: 001006; U6: 001973; miR-27a-5p: 002445; miR-146a-5p: 000468) on the same instrument. The gene and miRNA expression levels were conducted using the 2^−ΔΔCT^ method.

## 3. Results

**Figure 1:**
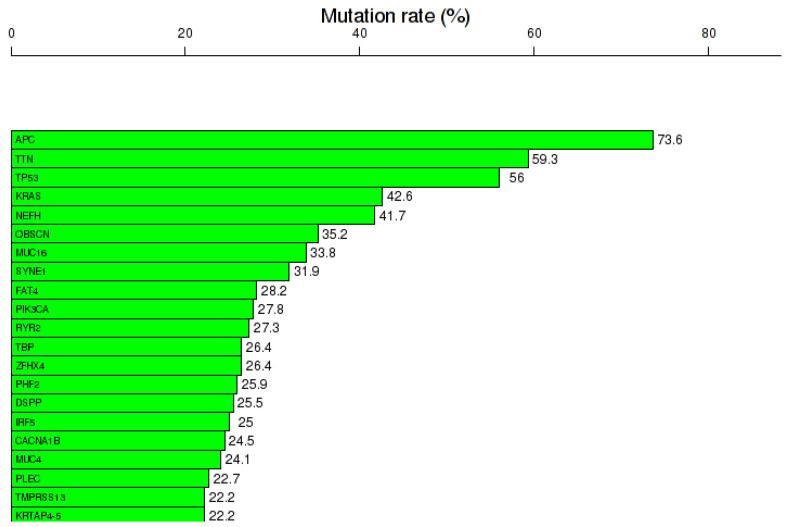
Mutational landscape, including the top 20 frequented mutated genes in COAD, mutation rates expressed as % from the total number of samples, generated using the online portal TCOA.

COAD reveals a specific gene expression pattern. Gene expression analysis was done using TCOA public database, revealing 1628 altered genes (363 overexpressed and 1265 downregulated genes), selecting a cut-off value |Fold change| >2 and FDR *q*-value ≤ 0.05 (Table 1). Additionally, the top 20 most frequent mutations are displayed in Figure 1.

EMT-specific mechanisms in COAD. EMT is the key mechanism involved in many solid tumors including COAD. EMT activation is connected with an increased metastatic rate and resistance to therapy, contributing to a poor prognosis. The NCBI list for specific transcripts related to EMT mechanisms was downloaded and was then overlapped with the altered genes, emphasizing common EMT-altered transcripts in a Venn diagram.

**Figure 2:**
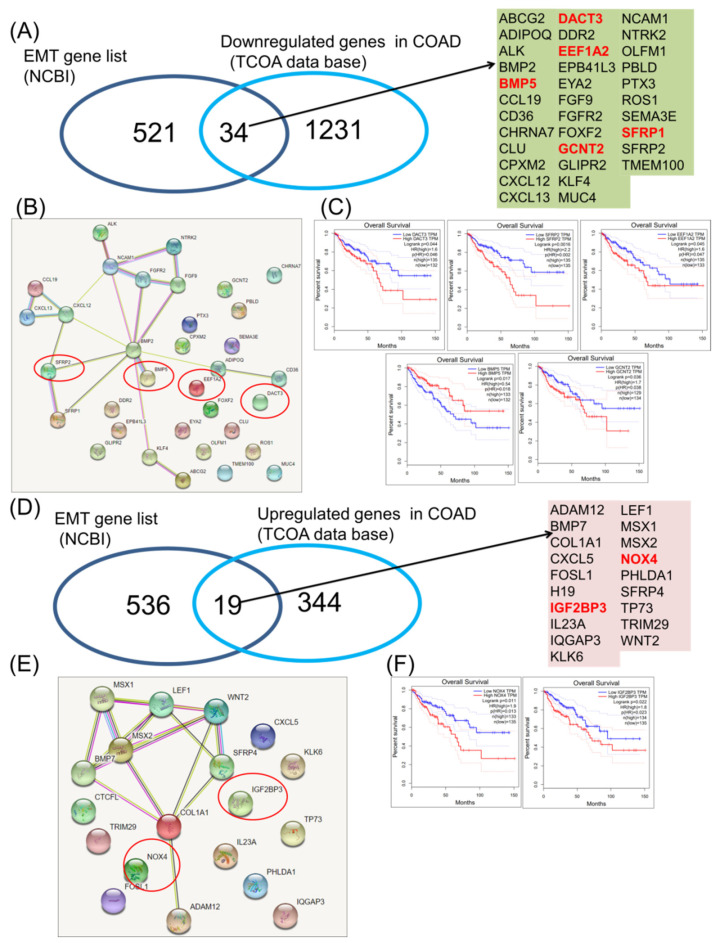
Common EMT downregulated and upregulated gene expression signature in COAD. (**A**) Venn diagram used to shows the common signature among the EMT gene list (downloaded from NCBI) and the downregulated genes in COAD, red letters—genes predict overall survival; (**B**) interaction network using String software for 34 downregulated genes common with EMT mechanisms, red circles—genes that predict overall survival outcomes; (**C**) COAD downregulated genes involved in EMT (DACT3, EEF1A2, BMP5, GCNT2 and SFRP1) predicting overall survival; (**D**) Venn diagram used to show the common signature among the EMT gene list (downloaded from NCBI) and the upregulated genes in COAD, red letters—genes predict overall survival; (**E**) interaction network using String software for 19 overexpressed genes common with EMT mechanisms, red circles—genes predict overall survival outcomes; (**F**) COAD overexpressed genes involved in EMT (NOX4 and IGF2BP3) predicting overall survival.

Regarding the downregulated genes, 34 of them display a common signature with EMT (Figure 2A), presented as a network (Figure 2B). Emphasis was to a high degree on interconnection among a part of them; BMP5, DACT3, EEF1A2, GCNT2 and SFRP1 were statistically significantly correlated with overall survival (Figure 2C), part of the gene network being BMP5 and SFRP1. Then, in the case of upregulated genes, 19 were found to be common, but only two of them (NOX4 and IGF2BP3) were correlated with the overall survival rate (Figure 2D).

**Figure 3:**
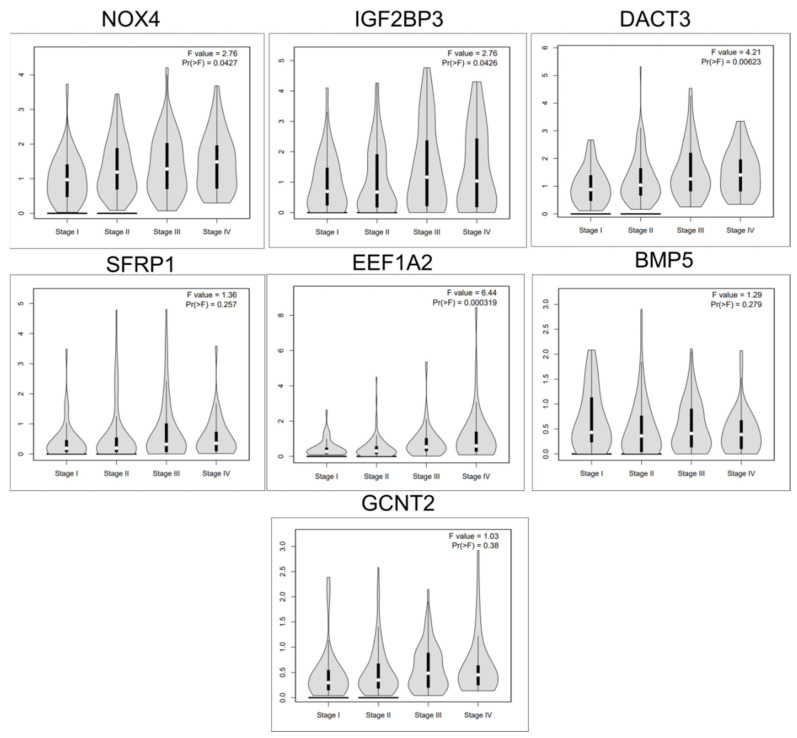
The expression level of NOX4, IGF2BP3, DACT3, SFRP1, BMP5, EEF1A2 and GCNT2 related to tumor stages in COAD, using GEPIA online tool.

Additionally, for the key EMT genes that predict overall survival rate, two overexpressed genes (NOX4 and IGF2BP3) and five downregulated genes (DACT3, EEF1A2, BMP5, GCNT2 and SFRP1) were represented, the expression level being displayed according to the stage of the disease, using online dataset analysis GEPIA, as shown in Figure 3, associated with the pathological stage in the case of NOX4, IGF2BP3, BMP5, DACT3 and EEF1A2.

**Figure 4:**
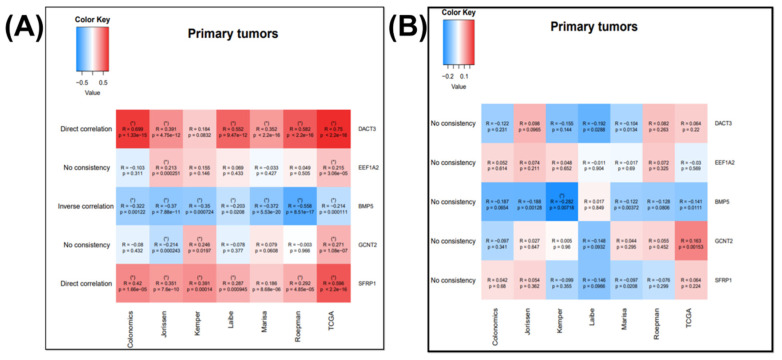
Correlation between the (**A**) NOX3 and EMT overexpressed genes, correlated with overall survival and (**B**) correlation between the IGF2BP3 and EMT overexpressed genes correlated with overall survival, generated using Cancertool (http://web.bioinformatics.cicbiogune.es/CANCERTOOL/, accessed on 22 April 2021) on five Affimetrix datasets (GSE44076, GSE14333, GSE33113, GSE37892, GSE39582), one Agilent dataset (GSE42284) and one RNAseq dataset. The color code shows the correlation status between the nominated gene pairs, red being toward 1 and blue toward −1. (* *p* ≤ 0.05 and correlation coefficient higher than 0.2 for direct and lower than −0.2 for inverse correlations).

To study the gene expression, and for the correlation among EMT genes, Cancertool [32] was used, as can be observed in Figure 4A for the NOX3 and EMT overexpressed genes and in Figure 4B for the IGF2BP3 and EMT overexpressed genes. The plot gene-to-gene correlations were calculated in the COAD datasets, presenting the type of correlation. This allowed the selection of the key EMT-correlated genes that uncover functional implications in cancer; this is the case of direct correlation of NOX4 with DACT3 and SFRP1 and inverse correlation of NOX4 and BMP5.

**Figure 5:**
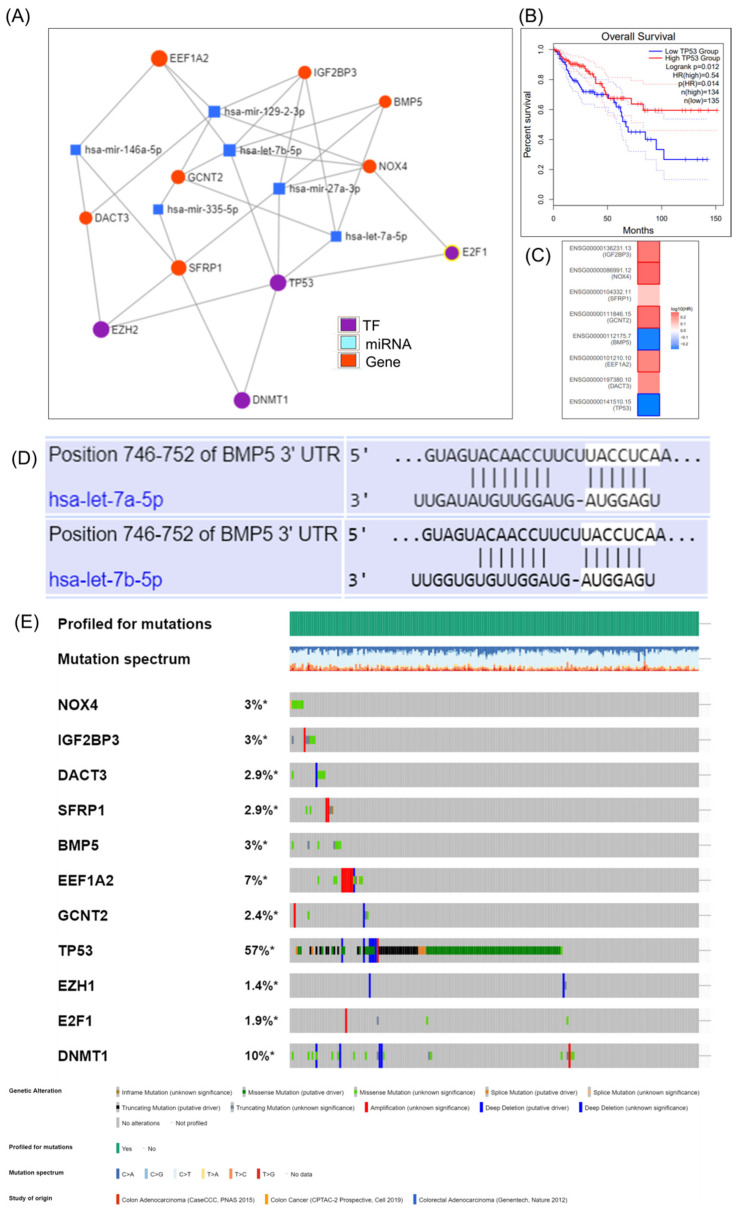
The target gene–miRNA interaction in COAD as revealed by the use of miRNET miRNA target gene online tool. (**A**) Interconnection between the EMT genes, key transcription factors and miRNA, generated using miRNET; (**B**) TP53 predicts overall survival rate in COAD; (**C**) survival map generated using GEPIA online tool; (**D**) prediction of has-let-7a-5p and let-7b-5p binding to BMP5 3′UTRs, generated using TargetScan 3.0 (http://www.targetscan.org/mamm_30/docs/help.html, accessed on 22 April 2021); (**E**) analysis of genetic alterations in COAD using cBioPortal data; online plot presents the mutation frequency for the selected genes found in RNAseq data. * Not Appliable.

miRNA-EMT gene interaction is shown in Figure 5, emphasizing a direct connection between DCAT3 and EEF1A2 with the TP53, one of the most frequent mutated gene in COAD, and also DNMT1 (methyl transferase), a frequent methylated gene in cancer, and two important transcription factors (EZH2 and E2F1). miRNET targeted genes analysis, showing the EMT genes targeted by key miRNAs, but none of the miRNAs predict the overall survival rate (Figure 5B). The additional mutational pattern displayed in Figure 5C was generated using cBioPortal online tool.

**Figure 6:**
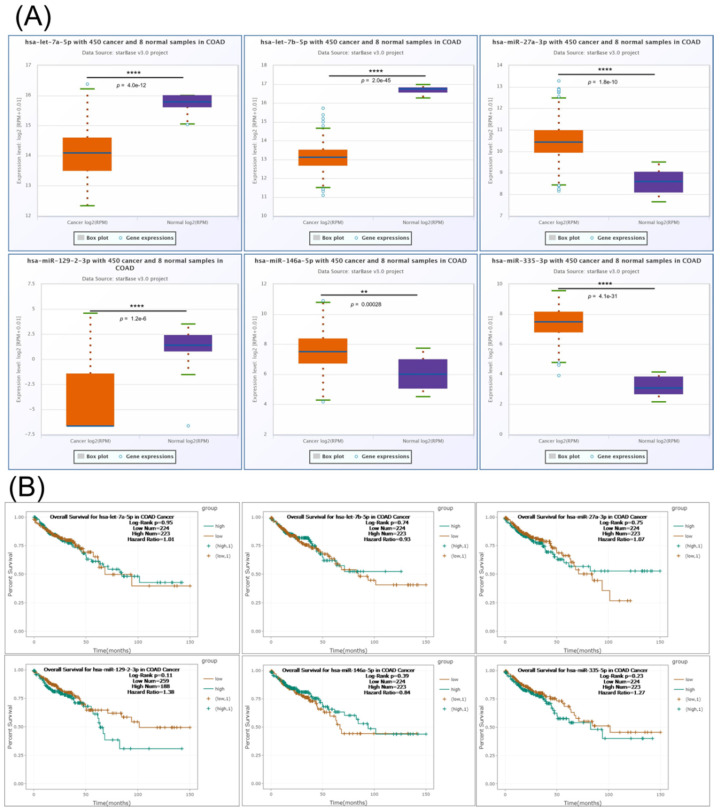
The expression level and survival analysis of miRNAs targeting key EMT genes in COAD with StarBase. (**A**) Expression levels in COAD of let-7a-5p, let-7b-5p, miR-27a-3p, miR-129-2-3p, miR-146a-5p and miR-335-3p based on StarBase; (**B**) Kaplan–Mayer plot in COAD for let-7a-5p, let-7b-5p, miR-27a-3p, miR-129-2-3p, miR-146a-5p and miR-335-3p based on StarBase. ns *p* > 0.05; ** *p* ≤ 0.01; **** *p* ≤ 0.0001.

Additional expression levels for the interconnected miRNAs transcript were done using StarBase online tool, revealing let-7a-5p, let-7b-5p and miR-129-2-3p downregulation, respectively, miR-27a-3p, miR-146a-5p and miR-335-3p overexpression (Figure 6A); none of these transcripts were able to predict overall survival rate (Figure 6B).

**Figure 7:**
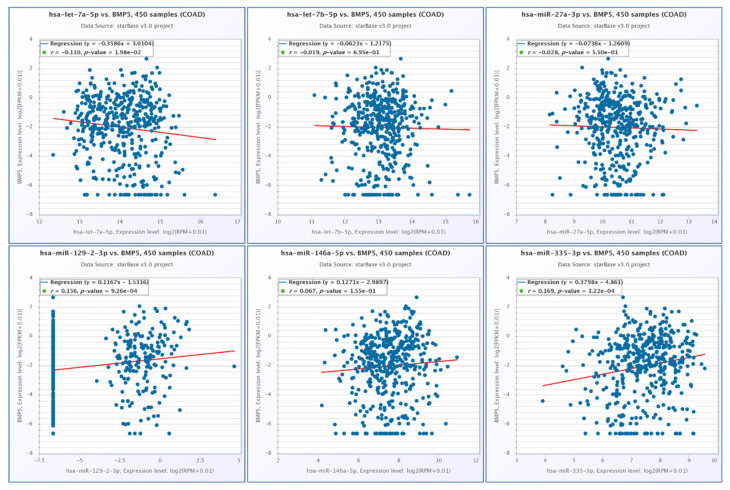
Correlation among the expression levels BMP5 and let-7a-5p, let-7b-5p, miR-27a-3p, miR-129-2-3p, miR-146a-5p and miR-335-3p in COAD, generated using StarBase.

Using StarBase, we observed a negative correlation between BMP5 and let-7a-5p, a direct target for BMP5, respectively, and a positive correlation between BMP5 and miR-129-2-3p and miR-335-3p, the two transcripts being indirectly connected in the interaction network. The BMP5 correlation with the selected miRNAs is presented in Figure 7.

● **Validation of BMP5 Genes by qRT-PCR**

**Figure 8:**
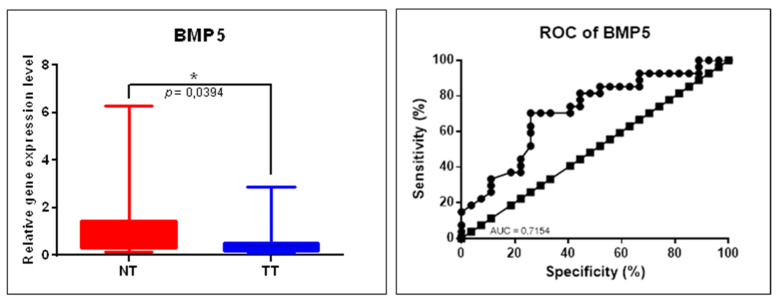
BMP5 gene expression alteration in COAD evaluated by qRT-PCR. Scatter plots demonstrate the downregulation of BMP5 in tumor tissues (TT, *n* = 29) versus normal tissues (TN, *n* = 29), B2M was used as a housekeeping gene (* *p* ≤ 0.05); ROC curves for each selected gene’s specificity and sensitivity (NT: normal tissue, TT: tumoral tissue, ROC: receiver-operating characteristic, AUC: area under ROC curve).

In order to further validate the gene expression alteration in COAD revealed using the GEPIA online tool, we performed qRT-PCR for BMP5; B2M gene was used as a housekeeping gene. Gene expression analysis found downregulation of BMP5 in tumor tissues versus normal adjacent tissues (Figure 8); the data are in agreement with those from the GEPIA database. An additional receiver-operating characteristic (ROC) curve was generated to evaluate the sensitivity and specificity of these genes; the AUC for BMP5 was 0.7154.

● **Validation of Key EMT miRNAs by qRT-PCR**

**Figure 9:**
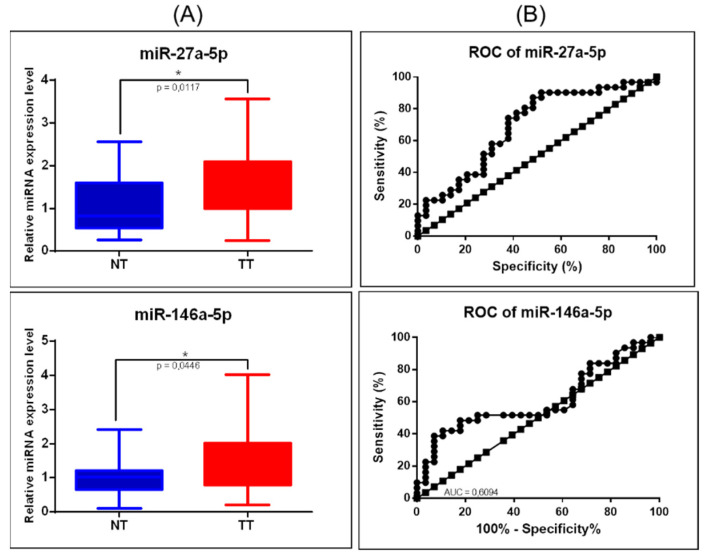
miRNA targeting key EMT signaling genes in COAD. (**A**) Scatter plots demonstrate the upregulation of miR-27a-5p and downregulation of miR-146a-5p in tumor tissues versus normal adjacent tissues; U6 and RNU48 were used as normalizations (* *p* ≤ 0.05); (**B**) ROC curve for miR-27a-5p and miR-146a-5p (NT: normal tissue, TT: tumoral tissue, ROC: receiver-operating characteristic, AUC: area under the curve).

As an additional validation step, from the mRNA-miRNA network, miR-27a-3p and miR-146a-5p were selected; U6 and RNU48 were used as housekeeping transcripts, with the data being analyzed using the ΔΔCt method, revealing overexpression of miR-27a-5p and miR-146a-5p (Figure 9A). Both evaluated transcripts were proved to be overexpressed in COAD, the higher AUC value being 0.6947 for miR-27a-5p (Figure 9B).

## 4. Discussion

Alteration on EMT-associated gene expression profiles was observed to be cell type specific and correlated with the degree of progression towards mesenchymal differentiation [13,33]. Therefore, EMT gene signature may act as a prognostic biomarker in COAD, as revealed by multiomics data [33]. EMT rendering was resistant not only to chemotherapy, but also to immunotherapies [34]. EMT directly regulates the expression of PD-L1 and is associated with several other checkpoint ligands, therefore promoting checkpoint-dependent resistance to anti-tumor immunity [34].

In the present study, a robust EMT gene signature, clinically significant to the patients with COAD, was identified to predict survival rate. The EMT signature proved to have prognostic effects, as was observed by the overall survival analysis using GEPIA for EMT genes.

EMT is a key biologic mechanism connected to decrease cell adhesion and to increase invasiveness; therefore, it is a key component for metastasis and drug resistance in many cancers, including COAD [19,33]. EMT is a complex regulatory mechanism that affects not only the expression of the epithelial proteins, but it is also related to important alteration on cytoskeleton architecture, as can be observed by the gene enrichment analysis presented in Figure 2 and Figure 3.

NOX4 is presented in the literature as a therapeutic target in digestive cancer [35], including in colorectal cancer [36], being correlated among others with VEGF, MAPK and PI3K/AKT [35]. NOX4 inhibition promotes the immunotherapy response by overcoming cancer-associated fibroblast-mediated CD8 T-cell exclusion [37]. Additionally, NOX4 is a key element in TGFβ and SMAD3-driven activation of EMT and migration of epithelial cells [38]. NOX family is overexpressed in colorectal cancer and correlated with the patient’s prognostic [36]. NOX4 is related not only to cell proliferation and apoptosis, but also with migration and metastasis [36,39].

Other studies present NOX4 and ITGA3 as relapse risk markers with important clinical interest, in order to understand how the mechanism of tumor’s progression to metastasis [40] is activated. An increased expression level for IGF2BP3 was correlated with aggressive phenotypes of colorectal cells [41,42,43]. IGF2BP3 is overexpressed in COAD samples [44], being related with adverse clinical outcome [43]. IGF2BP3 being presented as therapeutic target, especially for immunotherapy [41,43,44].

DACT3 is underexpressed in COAD and is an epigenetic regulator of Wnt/β-catenin, this gene was proven to be a prognostic factor for colorectal cancer. These emphasize the important role of epigenetic events on the modulation of response to therapy [45]. DACT3 was proven to be one of the key six hub genes related with prognostics and validated to be connected with the pathological stage in COAD [46].

The elongation factors, including EEF1A2, were studied in different cancer types, in COAD being downregulated, literature presenting them as biomarkers and therapeutic drug targets. BMP5 has an essential function in COAD initiation and development, as it is a tumor suppressor gene mutated in around 7% of the cases [47].

BMP5 is not in the top 20 frequently mutated genes, but is overexpressed in tumor tissue and correlated with overall survival rate, as our data showed. In spite of this, a previous study reveals that BMP5 genetic alteration in COAD is distinctive, and loss of BMP5 expression may be a COAD-specific event [47]. The BMP5 expression level was significantly decreased in tumors compared to adjacent normal tissues in TCGA cohort; mRNA expression level for this gene was also validated in our patient cohort. BMP5 is considered as an early event in colorectal cancer, with prognostic value, related with a coexpression pattern with E-cadherin and could be considered tissue-specific [47]. BMP5 belongs to the TGF-β/Smad signaling pathway; therefore, BMP5 expression was positively associated with epithelial markers and negatively associated with mesenchymal markers [47]. Additionally, BMP5 was proved to interact with PI3K-AKT and MAPKs signaling [48]. In our case, BMP5 was inversely correlated with NOX4 and let-7a-5p and positively correlated with miR-129-2-3p and miR-335-3p (Figure 4 and Figure 7).

Recent studies demonstrated that epigenetic alterations also play important roles in EMT [49,50]. Our study reveals a connection between the epigenetic mechanisms and EMT, with an emphasis on a direct interconnection between the EMT gene predicting overall survivals and DNMT1 (Figure 6), a key gene involved in the epigenetic mechanism. Other EMT-related genes with prognostic value are represented by GCNT2 [51], retrieved in the literature as methylated, correlated with lymph node metastasis of colorectal cancer [51,52].

Although the detailed role of EMT in metastatic cascade, especially in COAD, remains poorly understood [53], the alterations in the miRNA regulatory network are essential for the activation of EMT [11,54,55]. miR-27b-3p is overexpressed in COAD, as the qRT-PCR data display. A previous study revealed that miR-27b-3p is a potential indicator of efficiency of chemotherapy and a therapeutic target [56]. This transcript was proven to promote migration and invasion in colorectal cancer [57]. miR-146a-5p proved to have a dual role in metastasis and disease progression [58]. In our patient cohort, it was found to be overexpressed in tumor tissue versus normal tissue in COAD. Other studies observed an overexpression in colon cancer cells, being related to induced immune suppression and drug resistance, not only to EMT [59]. This is the case of let-7a that is related to suppressing antitumor immunity, being proposed as a potential target of immunotherapy in COAD [60]. Another study reveals that let-7a and let-7b expression is dependent on TP53, a gene frequently mutated in COAD [61] and having an important role in cancer [62].

## 5. Conclusions

In conclusion, our results showed that the EMT is not only a cellular tool for reacting to environmental changes and resistance to them, particularly those related to treatment response, but also related to patient prognosis in COAD. Our study reveals regulatory interactions between the EMT gene and miRNAs. The identified EMT signature was proved to interact with key signaling pathways, sustaining tumor progression; therefore, these genes can be considered not only as prognostic markers, but also as therapeutic targets. Additionally, it is tempting to speculate that several of the identified miRNAs can potentially serve as biomarkers with clinical implication. These results need further validation in a more enlarged study on an additional group of patients with COAD, to confirm the capacity to convey prognostic information in a pre-treatment setting.

## Figures and Tables

**Table 1:** Clinical data of COAD patients used qRT-PCR validation of BMP5, miR-27a-5p and miR-146a-5p.

Sample	Age	Sex	TNM Status
1	84	M	T3N2bM1
2	37	M	T4aN0
3	68	F	T3N1b
4	59	M	T3N0
5	71	M	T3N0Mx
6	75	F	T3N2a
7	78	M	T3N0
8	82	M	T2N0
9	69	M	T3N1aMx
10	68	M	T4bN2bM1
11	81	F	T3N2a
12	62	M	T3N1a
13	49	F	T3N1b
14	61	F	T3N1aMx
15	83	M	T3N2bM1
16	80	F	T3N1a
17	68	F	T3N2
18	78	M	T3N0
19	79	F	T3N1aMx
20	61	F	T2N0
21	55	F	T1N0
22	68	M	T3N1cMx
23	59	M	T3N1a
24	79	M	T3N2a
25	66	M	T4aN0
26	71	M	T3N2
27	57	F	T3N1aMx
28	80	F	T2N0
29	77	M	T3N1a

## Data Availability

Additional data that support the findings of this study are available on request from the corresponding author.
